# Supplementary material for: Modeling glioblastoma heterogeneity as a dynamic network of cell states
Source: Mol Syst Biol. 2021 Sep 16;17(9):e10105. doi: 10.15252/msb.202010105 (PMC8444284; doi:10.15252/msb.202010105)
Supplement: Supplementary file 6 — Source Data for Figure 5 [file MSB-17-e10105-s004.zip › Figure5A_sourcedata/GSEA_3071/nosigdb_stateII.GseaPreranked.1622795151171/NB_KI_CLUSTER2.html]

Details for gene set NB\_KI\_CLUSTER2[GSEA]

|  || Dataset | state23071 |
| Phenotype | NoPhenotypeAvailable |
| Upregulated in class | na\_neg |
| GeneSet | NB\_KI\_CLUSTER2 |
| Enrichment Score (ES) | -0.5135247 |
| Normalized Enrichment Score (NES) | -3.0927317 |
| Nominal p-value | 0.0 |
| FDR q-value | 0.0 |
| FWER p-Value | 0.0 |
Table: GSEA Results Summary

  

Fig 1: Enrichment plot: NB\_KI\_CLUSTER2      
 Profile of the Running ES Score & Positions of GeneSet Members on the Rank Ordered List

  

| PROBE | GENE SYMBOL | GENE\_TITLE | RANK IN GENE LIST | RANK METRIC SCORE | RUNNING ES | CORE ENRICHMENT || 1 | RAB3B |  |  | 84 | 1.107 | -0.0076 | No |
| 2 | ALCAM |  |  | 288 | 0.771 | -0.0495 | No |
| 3 | CNRIP1 |  |  | 442 | 0.655 | -0.0801 | No |
| 4 | MEST |  |  | 563 | 0.593 | -0.1032 | No |
| 5 | RAP1GAP2 |  |  | 602 | 0.577 | -0.1057 | No |
| 6 | AHI1 |  |  | 814 | 0.494 | -0.1530 | No |
| 7 | TLN2 |  |  | 917 | 0.467 | -0.1731 | No |
| 8 | H1F0 |  |  | 1229 | 0.391 | -0.2470 | No |
| 9 | TTL |  |  | 1289 | 0.380 | -0.2572 | No |
| 10 | EFNB2 |  |  | 1306 | 0.377 | -0.2566 | No |
| 11 | SESTD1 |  |  | 1613 | 0.321 | -0.3301 | No |
| 12 | SHC1 |  |  | 1671 | 0.314 | -0.3407 | No |
| 13 | CEP290 |  |  | 1716 | 0.308 | -0.3480 | No |
| 14 | TBCD |  |  | 1868 | 0.290 | -0.3827 | No |
| 15 | UBE2E3 |  |  | 1896 | 0.286 | -0.3860 | No |
| 16 | STX2 |  |  | 1926 | 0.282 | -0.3898 | No |
| 17 | PKIB |  |  | 2132 | 0.259 | -0.4385 | No |
| 18 | EIF2AK2 |  |  | 2148 | 0.257 | -0.4391 | No |
| 19 | C5orf24 |  |  | 2219 | -0.250 | -0.4538 | No |
| 20 | DOCK4 |  |  | 2230 | -0.251 | -0.4532 | No |
| 21 | TSPYL4 |  |  | 2235 | -0.252 | -0.4511 | No |
| 22 | GNAI1 |  |  | 2254 | -0.254 | -0.4525 | No |
| 23 | PCBD1 |  |  | 2255 | -0.254 | -0.4494 | No |
| 24 | DYNC1I2 |  |  | 2260 | -0.255 | -0.4473 | No |
| 25 | ZC2HC1A |  |  | 2277 | -0.257 | -0.4482 | No |
| 26 | PPP2CB |  |  | 2394 | -0.270 | -0.4742 | No |
| 27 | CHST12 |  |  | 2421 | -0.273 | -0.4774 | No |
| 28 | VEZT |  |  | 2424 | -0.273 | -0.4745 | No |
| 29 | EXOC4 |  |  | 2431 | -0.275 | -0.4727 | No |
| 30 | MTMR4 |  |  | 2496 | -0.285 | -0.4853 | No |
| 31 | GALNT11 |  |  | 2498 | -0.285 | -0.4821 | No |
| 32 | PRH1 |  |  | 2504 | -0.286 | -0.4798 | No |
| 33 | NUDT3 |  |  | 2506 | -0.286 | -0.4766 | No |
| 34 | SRGAP3 |  |  | 2508 | -0.286 | -0.4733 | No |
| 35 | CLASP2 |  |  | 2514 | -0.287 | -0.4710 | No |
| 36 | RSBN1L |  |  | 2546 | -0.293 | -0.4752 | No |
| 37 | DTX3 |  |  | 2572 | -0.297 | -0.4779 | No |
| 38 | YPEL1 |  |  | 2589 | -0.299 | -0.4783 | No |
| 39 | MARCH6 |  |  | 2612 | -0.303 | -0.4801 | No |
| 40 | PLEKHA8 |  |  | 2614 | -0.303 | -0.4766 | No |
| 41 | PREPL |  |  | 2621 | -0.304 | -0.4744 | No |
| 42 | PCBP4 |  |  | 2633 | -0.306 | -0.4734 | No |
| 43 | TMX4 |  |  | 2693 | -0.315 | -0.4845 | No |
| 44 | CEP170 |  |  | 2737 | -0.324 | -0.4914 | No |
| 45 | TSHZ2 |  |  | 2764 | -0.329 | -0.4939 | No |
| 46 | RALGDS |  |  | 2775 | -0.331 | -0.4923 | No |
| 47 | SECISBP2 |  |  | 2802 | -0.335 | -0.4948 | No |
| 48 | RNMT |  |  | 2805 | -0.336 | -0.4912 | No |
| 49 | MAPRE3 |  |  | 2863 | -0.346 | -0.5013 | No |
| 50 | TRIM13 |  |  | 2869 | -0.348 | -0.4983 | No |
| 51 | ZNF292 |  |  | 2882 | -0.352 | -0.4970 | No |
| 52 | ZC3H6 |  |  | 2884 | -0.352 | -0.4929 | No |
| 53 | PRAF2 |  |  | 2918 | -0.359 | -0.4968 | No |
| 54 | TMOD2 |  |  | 2985 | -0.370 | -0.5090 | Yes |
| 55 | MXI1 |  |  | 3000 | -0.372 | -0.5079 | Yes |
| 56 | TANC2 |  |  | 3015 | -0.378 | -0.5068 | Yes |
| 57 | TP53BP1 |  |  | 3024 | -0.378 | -0.5042 | Yes |
| 58 | HIPK2 |  |  | 3029 | -0.379 | -0.5005 | Yes |
| 59 | ATXN7L3B |  |  | 3040 | -0.382 | -0.4983 | Yes |
| 60 | KANSL1 |  |  | 3045 | -0.384 | -0.4946 | Yes |
| 61 | SCRN1 |  |  | 3051 | -0.386 | -0.4911 | Yes |
| 62 | AKAP9 |  |  | 3066 | -0.389 | -0.4899 | Yes |
| 63 | LAPTM4B |  |  | 3068 | -0.389 | -0.4853 | Yes |
| 64 | IFI27L2 |  |  | 3071 | -0.390 | -0.4810 | Yes |
| 65 | SLC25A36 |  |  | 3080 | -0.393 | -0.4782 | Yes |
| 66 | WDR6 |  |  | 3113 | -0.402 | -0.4813 | Yes |
| 67 | CHD9 |  |  | 3132 | -0.409 | -0.4808 | Yes |
| 68 | ENAH |  |  | 3136 | -0.410 | -0.4765 | Yes |
| 69 | TUT4 |  |  | 3144 | -0.413 | -0.4732 | Yes |
| 70 | PNISR |  |  | 3160 | -0.416 | -0.4719 | Yes |
| 71 | ARMCX1 |  |  | 3188 | -0.425 | -0.4735 | Yes |
| 72 | BRI3 |  |  | 3242 | -0.438 | -0.4815 | Yes |
| 73 | PLD3 |  |  | 3285 | -0.452 | -0.4865 | Yes |
| 74 | EIF4A2 |  |  | 3289 | -0.453 | -0.4817 | Yes |
| 75 | SMARCA1 |  |  | 3293 | -0.455 | -0.4769 | Yes |
| 76 | ORAI2 |  |  | 3297 | -0.456 | -0.4720 | Yes |
| 77 | ADCY1 |  |  | 3299 | -0.457 | -0.4666 | Yes |
| 78 | CASC15 |  |  | 3308 | -0.460 | -0.4630 | Yes |
| 79 | ANKRD12 |  |  | 3311 | -0.460 | -0.4578 | Yes |
| 80 | UBN2 |  |  | 3317 | -0.463 | -0.4534 | Yes |
| 81 | GSTA4 |  |  | 3318 | -0.463 | -0.4477 | Yes |
| 82 | SLC39A10 |  |  | 3320 | -0.463 | -0.4422 | Yes |
| 83 | IFT20 |  |  | 3337 | -0.469 | -0.4405 | Yes |
| 84 | BRD3 |  |  | 3346 | -0.474 | -0.4366 | Yes |
| 85 | TSPYL1 |  |  | 3353 | -0.479 | -0.4322 | Yes |
| 86 | CASD1 |  |  | 3357 | -0.480 | -0.4271 | Yes |
| 87 | PPP1R9A |  |  | 3366 | -0.486 | -0.4231 | Yes |
| 88 | MARCKS |  |  | 3369 | -0.487 | -0.4176 | Yes |
| 89 | PTPRA |  |  | 3392 | -0.494 | -0.4171 | Yes |
| 90 | TULP4 |  |  | 3398 | -0.497 | -0.4122 | Yes |
| 91 | APC |  |  | 3422 | -0.510 | -0.4118 | Yes |
| 92 | TAOK1 |  |  | 3442 | -0.516 | -0.4102 | Yes |
| 93 | KIDINS220 |  |  | 3459 | -0.523 | -0.4078 | Yes |
| 94 | NOL4L |  |  | 3464 | -0.525 | -0.4024 | Yes |
| 95 | ZNF397 |  |  | 3498 | -0.540 | -0.4041 | Yes |
| 96 | MAP1LC3A |  |  | 3501 | -0.542 | -0.3979 | Yes |
| 97 | SLC39A6 |  |  | 3502 | -0.543 | -0.3912 | Yes |
| 98 | ATRX |  |  | 3509 | -0.546 | -0.3860 | Yes |
| 99 | BPTF |  |  | 3510 | -0.547 | -0.3792 | Yes |
| 100 | ARHGAP21 |  |  | 3538 | -0.564 | -0.3791 | Yes |
| 101 | GNG2 |  |  | 3543 | -0.567 | -0.3731 | Yes |
| 102 | RETREG1 |  |  | 3555 | -0.573 | -0.3689 | Yes |
| 103 | ATP6V1B2 |  |  | 3557 | -0.574 | -0.3620 | Yes |
| 104 | EFHC1 |  |  | 3566 | -0.581 | -0.3569 | Yes |
| 105 | NPDC1 |  |  | 3573 | -0.585 | -0.3512 | Yes |
| 106 | ZEB1 |  |  | 3589 | -0.598 | -0.3476 | Yes |
| 107 | FOXO3 |  |  | 3594 | -0.601 | -0.3412 | Yes |
| 108 | FNBP1L |  |  | 3615 | -0.614 | -0.3387 | Yes |
| 109 | VAT1 |  |  | 3623 | -0.619 | -0.3329 | Yes |
| 110 | MUM1 |  |  | 3632 | -0.629 | -0.3272 | Yes |
| 111 | DPYSL2 |  |  | 3645 | -0.642 | -0.3223 | Yes |
| 112 | TET1 |  |  | 3646 | -0.643 | -0.3144 | Yes |
| 113 | FOXN3 |  |  | 3671 | -0.661 | -0.3123 | Yes |
| 114 | IP6K2 |  |  | 3689 | -0.672 | -0.3083 | Yes |
| 115 | MCF2L |  |  | 3691 | -0.673 | -0.3003 | Yes |
| 116 | MTURN |  |  | 3693 | -0.677 | -0.2922 | Yes |
| 117 | GOLM1 |  |  | 3699 | -0.684 | -0.2850 | Yes |
| 118 | PCDH9 |  |  | 3707 | -0.691 | -0.2783 | Yes |
| 119 | THRA |  |  | 3717 | -0.698 | -0.2719 | Yes |
| 120 | SMAD1 |  |  | 3719 | -0.699 | -0.2636 | Yes |
| 121 | GSE1 |  |  | 3720 | -0.699 | -0.2549 | Yes |
| 122 | ZNF91 |  |  | 3729 | -0.708 | -0.2482 | Yes |
| 123 | CLCN3 |  |  | 3734 | -0.711 | -0.2405 | Yes |
| 124 | ZKSCAN1 |  |  | 3736 | -0.711 | -0.2320 | Yes |
| 125 | C16orf45 |  |  | 3771 | -0.762 | -0.2312 | Yes |
| 126 | MAPK10 |  |  | 3798 | -0.787 | -0.2281 | Yes |
| 127 | BICD1 |  |  | 3814 | -0.801 | -0.2220 | Yes |
| 128 | MSI2 |  |  | 3816 | -0.802 | -0.2123 | Yes |
| 129 | PSD3 |  |  | 3834 | -0.826 | -0.2065 | Yes |
| 130 | PFN2 |  |  | 3841 | -0.832 | -0.1977 | Yes |
| 131 | PGM2L1 |  |  | 3843 | -0.832 | -0.1877 | Yes |
| 132 | PRXL2A |  |  | 3849 | -0.841 | -0.1786 | Yes |
| 133 | CCDC88A |  |  | 3854 | -0.848 | -0.1692 | Yes |
| 134 | MPP6 |  |  | 3855 | -0.848 | -0.1587 | Yes |
| 135 | DAAM1 |  |  | 3856 | -0.849 | -0.1482 | Yes |
| 136 | TCEA2 |  |  | 3868 | -0.877 | -0.1402 | Yes |
| 137 | DSEL |  |  | 3897 | -0.940 | -0.1357 | Yes |
| 138 | PBX1 |  |  | 3914 | -0.977 | -0.1277 | Yes |
| 139 | KCNQ1OT1 |  |  | 3916 | -0.988 | -0.1158 | Yes |
| 140 | BAZ2B |  |  | 3947 | -1.046 | -0.1105 | Yes |
| 141 | NEDD4L |  |  | 3960 | -1.090 | -0.1001 | Yes |
| 142 | PJA2 |  |  | 3974 | -1.128 | -0.0895 | Yes |
| 143 | TCF4 |  |  | 3984 | -1.173 | -0.0773 | Yes |
| 144 | SESN3 |  |  | 4003 | -1.260 | -0.0663 | Yes |
| 145 | KDM5B |  |  | 4016 | -1.364 | -0.0525 | Yes |
| 146 | CLSTN1 |  |  | 4020 | -1.391 | -0.0361 | Yes |
| 147 | CLU |  |  | 4069 | -1.908 | -0.0248 | Yes |
| 148 | NRP2 |  |  | 4087 | -2.583 | 0.0028 | Yes |
Table: GSEA details [plain text format]

  

Fig 2: NB\_KI\_CLUSTER2: Random ES distribution      
 Gene set null distribution of ES for **NB\_KI\_CLUSTER2**
